# Plasma antibodies against heat shock protein 70 correlate with the incidence and severity of asthma in a Chinese population

**DOI:** 10.1186/1465-9921-6-18

**Published:** 2005-02-14

**Authors:** Miao Yang, Tangchun Wu, Longxian Cheng, Feng Wang, Qingyi Wei, Robert M Tanguay

**Affiliations:** 1 Institute of Occupational Medicine, Tongji Medical College, Huazhong University of Science and Technology, Wuhan 430030, China; 2 Union Hospital, Tongji Medical College, Huazhong University of Science and Technology, Wuhan 430022, China; 3 Laboratory of Cell and Developmental Genetics, Dept Medicine, Faculty of Medicine, Pav. C.E. Marchand, Université Laval, Québec, G1K 7P4, Canada

## Abstract

**Background:**

The heat shock proteins (Hsps) are induced by stresses such as allergic factors and inflammatory responses in bronchi epithelial cells and therefore may be detectable in patients with asthma. However, the etiologic link between anti-Hsps and asthma (its severity and related inflammatory responses such as interleukin-4 and immunoglobulin E) has not been established. We determined whether antibodies against Hsp60 and Hsp70 were present in patients with asthma and evaluated their associations with risk and severity of asthma.

**Methods:**

We determined the levels of anti-Hsp60 and anti-Hsp70 by immunoblot and their associations with risk and symptom severity of asthma in 95 patients with asthma and 99 matched non-symptomatic controls using multivariate logistic regression analysis.

**Results:**

Compared to the controls, asthma patients were more likely to have detectable anti-Hsp60 (17.2% vs 5.1%) and anti-Hsp70 (33.7% vs 8.1%) (p ≤ 0.001). In particular, the presence of anti-Hsp70 was associated with a greater than 2 fold risk for asthma (adjusted OR = 2.21; 95% CI = 1.35~3.59). Furthermore, both anti-Hsp60 and anti-Hsp70 levels were positively correlated with symptom severity (p < 0.05) as well as interleukin-4 and immunoglobulin E (p < 0.05). Individuals with antibodies against anti-Hsp60 and anti-Hsp70 were more likely to have a family history of asthma (p < 0.001) and higher plasma concentrations of total immunoglobulin E (p = 0.001) and interleukin-4 (p < 0.05) than those without antibodies.

**Conclusions:**

These data suggest that anti-Hsp60 and especially anti-Hsp70 correlate with the attacks and severity of asthma. The underlying molecular mechanisms linking antibodies to heat shock proteins and asthma remain to be investigated.

## Background

Heat shock proteins (Hsps) are highly conserved proteins inducible in response to a wide variety of stresses (such as exposure to heat) and pathological (viral, bacterial or parasitic infections, and inflammation) or physiological (growth factors, cell differentiation, and hormonal stimulation) stimuli [1,2]. There are six main Hsp families (i.e., Hsp110, Hsp90, Hsp/Hsc70, Hsp60, Hsp40, and Hsp10-30) categorized on the basis of their apparent molecular masses detected by sodium dodecyl sulphate polyacrylamide gel electrophoresis (SDS-PAGE). Hsps are involved in various biological functions including 1) intracellular chaperones of naive, aberrantly folded or mutated proteins, 2) cytokines of signal transduction cascades involved in inflammatory response, and 3) cytoprotective agents in response to the aforementioned stress stimuli [1,3,4]. In addition, Hsps are also involved in transport of proteins and peptides through cellular compartments, and can bind to endogenous antigenic peptides and transport them to the major histocompatibility complexes [5,6]. This suggests that Hsps may modulate immune and inflammatory responses and may be involved in the pathogenesis and/or be markers for risk and prognosis of certain diseases including asthma [7-13], given that many of the stress stimuli mentioned above are factors that can induce attacks of asthma.

Asthma is a multifactorial and likely multigenic immune inflammatory disease of the upper airways, arising from complex interactions among environmental and genetic factors [14,15]. These factors may induce Hsp60 and Hsp70 in bronchi epithelial cells during the development of asthma [16]. Some Hsps present as self-antigens to the immune system, resulting in the production of autoantibodies in patients with inflammatory diseases and immune disorders after infections by bacteria, mycobacteria and Chlamydia [17-19]. Studies have demonstrated that these autoantibodies against Hsps were involved in the pathogenesis and/or prognosis of some diseases [8,20-23].

Up to now, few studies investigated possible associations of autoantibodies to human Hsps with the severity of asthma. In the present study, we determined the presence of autoantibodies to human Hsp60 and Hsp70 in 193 subjects with (n = 95) and without (n = 99) asthma by immunoblot analysis, and evaluated the associations of these autoantibodies with asthma severity and their correlation with interleukin-4 (IL-4) and immunoglobulin E (IgE) both involved in the development of asthma, by using multivariate logistic regression analyses.

## Methods

### Subjects and groups

This 95 patients with asthma (54 males and 41 females) and 99 healthy, age-matched non-asthmatic controls (64 males and 35 females) were residents living in the same geographic area. Patients and controls were from Wuchang, one of the three cities of Wuhan and were all of Han nationality. Their age ranged from 10 to 45 years old (Table 1). All 95 patients were diagnosed according to diagnostic criteria and principles of management of asthma proposed by the American Thoracic Society [24] and did not have other pulmonary, cardiovascular and gastro-duodenal diseases. A standardized questionnaire was completed for each individual by physicians with extensive experience in allergic and immune diseases to obtain demographic information and known risk factors for asthma including personal and family history of asthma and frequency of attacks. Selection criteria for the controls included the absence of any personal history of asthma. Health examination and physical sign findings such as wheezing and forced expiratory volume (FEV%Pre) were also recorded. Neither patients nor controls had any history of chronic diseases such as cancer, diabetes, cardiovascular diseases and gastro-duodenal diseases. Patients with asthma were grouped by symptom severity and medication use according to the 2002 Global Initiative for Asthma Guidance [25] as intermittent, mild persistent, moderate persistent and severe persistent. Venous blood was collected into heparinized tubes to separate plasma for the detection of anti-Hsp60 and anti-Hsp70 as well as IgE and IL-4. Plasma samples from patients and controls were stored in aliquots at -80°C and thawed only once immediately before the tests were performed. Written informed consent was obtained from patients and controls, and the study was approved by the Tongji Medical College Ethics Committee.

**Table 1:** Comparison of selected variables between patients with asthma and healthy controls

	Patients with asthma (n = 95)	Control subjects (n = 99)	*P *value
Sex (M/F)	54/41	64/35	0.266
Age (years, mean ± SD)	28.2 ± 15.8	29.1 ± 13.9	0.680
Attack once a week or a day (yes/no)	95/95	0/99	<0.000
Sign (wheezing) (yes/no)	95/95	0/99	<0.000
FEV_1_%Pre	58.4 ± 18.9	96.5 ± 9.8	<0.001
IgE (IU/ml, mean ± SD)	486.9 ± 595.5	75.8 ± 124.9	<0.001
IL-4 (ng/L, mean ± SD)	31.7 ± 17.1	5.1 ± 3.8	<0.001

### Determination of anti-Hsp60 and anti-Hsp70

**Figure 1:**
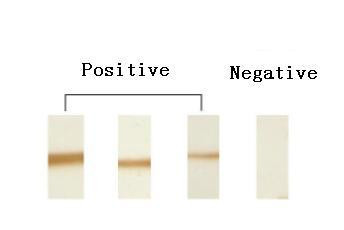
Purified recombinant Hsp70 was electrophoresed in SDS-PAGE, transferred to nitrocellulose membranes and cut into 2 – 3 mm wide strips. These were incubated with the plasma and the presence of antibodies to Hsp70 detected as described in Methods. Lane 1 – 3: positive; Lane 4: negative.

Recombinant human Hsp60 and inducible Hsp70 were obtained through the expression of corresponding cDNA in NaCl-induced *E. coli *GJ1168 cells using pET30 (Novegen) as the expression vector [26]. Approximately 10–15 μg of recombinant human Hsp60 or Hsp70 was loaded on each SDS-PAGE gel without combs, separated, and transferred by electrophoresis to nitrocellulose membranes. The band containing Hsp60 or Hsp70 was cut into 2 mm × 3 mm pieces and marked with a small red dot on the protein side of the membrane. These membrane pieces were placed in individual wells of an ELISA plate, rinsed with PBS, saturated with 100 μl of blocking buffer (PBS containing 5% skim milk powder) for 1 h at 37°C with gentle agitation and washed with PBS-0.05% Tween 80 for 5 min. The plasma diluted 1:10, 1:20, 1:40 and 1:80 in 100 μl PBS containing 5% skim milk powder was incubated with the membrane pieces at 37°C for 2 h with gentle agitation. After washing the membrane pieces six times (10 min each) with 200 μl PBS-0.05% Tween 80, 100 μl of HRP labelled goat anti-human IgG (Sigma) in blocking buffer (1:2500) was added and the incubation continued at 37°C for 1 h. The membrane pieces were washed again six times (10 min) with 200 μl PBS-0.05% Tween 80. The presence of anti-Hsp60 or anti-Hsp70 was then revealed with DAB (3,3-diaminobenzidine tetra hydrochloride) for 3–5 min. A visible brown band on the membrane piece was regarded as a positive test and a colourless membrane as a negative test [21,22]. An example of the microblot technique is shown in Figure 1. Samples were scored in a double blind manner by three different investigators.

### Detection of plasma IgE and IL-4

Total IgE was measured in plasma by using a fluorescence enzyme immunoassay kit from Bayer Company (Leverkusen, Germany). IL-4 was determined using a commercial enzyme-linked immunosorbent assay kit from OptEIA (Pharmingen, California, U.S.A). Each sample was tested in duplicate by a series of dilutions using a standard provided with the kit.

### Statistical analyses

All the continuous data (e.g., age, FEV1, IGE, IL4) were presented as the mean ± standard deviation (SD) and analysed by the Student's *t *test. Frequency data (e.g., sex) were analysed by the Chi-square test. The associations were estimated by fitting univariate and multivariate logistic regression models. Statistical inferences were based on a significance level of 0.05. All analyses were two-sided and performed by using the Statistical Package for Social Sciences (SPSS) software (Version, Chicago).

## Results

The patient and control groups comprised 56.8% and 64.6% of males, respectively, and had mean ages of 28.2 and 29.1 years, respectively (Table 1). All patients had regular asthma attacks and sign of wheezing, while none of the controls showed any of these signs. Asthma patients had a significantly lower FEV1%Pre than the controls (58.4 vs 96.5, p < 0.001). In addition, the asthma patients had significantly higher concentrations of total IgE and IL-4 than the controls (p < 0.001 for all comparisons).

### Presence of anti-Hsp60 and anti-Hsp70 in plasma

We first looked for the presence of anti-Hsp60 and anti-Hsp70 in plasma at dilutions of 1:10 to 1:80 in the patients with asthma and in the matched controls. At a dilution of 1:10, asthma patients had a significantly higher positive rate of anti-Hsp60 than the controls (17.9% vs 5.1%, p = 0.001). In the case of Hsp70, antibodies were observed in 33.7% of patients as compared to 8.1% in the controls at a dilution of 1:10 (p < 0.001). At plasma dilutions between 1:20 to 1:80, the difference in the detection rates of both anti-Hsp60 and anti-Hsp70 between the patients and controls remained highly significant (Table 2). The combined detection rate of both anti-Hsp60 and anti-Hsp70) at the lower plasma dilution (1:10) was also globally much higher in the asthma patients (38.9%) than in the controls (9.1%).

**Table 2:** Comparison of positive rates of different titers for ant-Hsp60 and anti-Hsp70 in plasma of patients with asthma and healthy controls

	Titers	Patients with asthma (n = 95)	Control subjects (n = 99)	*P *value
		n (%)	n (%)	
Anti-Hsp60	1:10	17 (17.9)	5 (5.1)	0.001
	1:20	8 (8.4)	1 (1.0)	0.014
	1:40	5 (5.3)	0 (0.0)	0.027
	1:80	5 (5.3)	0 (0.0)	0.027

Anti-Hsp70	1:10	32 (33.7)	8 (8.1)	<0.001
	1:20	19 (20.0)	4 (4.0)	<0.001
	1:40	13 (13.7)	1 (1.0)	<0.001
	1:80	9 (9.5)	0 (0.0)	0.001

Anti-Hsps*	1:10	37 (38.9)	9 (9.1)	<0.001

* Combined positive rate of anti-Hsp60 and /or anti-Hsp70 for titers 1:10–1:80.

### Association between anti-Hsp60 and anti-Hsp70 with risk for asthma

Further analysis for asthma risk factors (sex, age, family history) and anti-Hsp60 and anti-Hsp70 was carried out by using a multivariate logistic regression model built with a forward stepwise selection procedure (p values for entry and removal, 0.10) and also based on clinical experience. The results in Table 3 show a statistically significant positive association between the presence of anti-Hsp70 and risk for asthma (p = 0.001), representing a greater than 2-fold increased risk for asthma (adjusted OR = 2.21; 95% CI = 1.35~3.59) (Table 3). However, no significant association of anti-Hsp60 with risk for asthma was found (p = 0.161).

**Table 3:** Multivariate logistic regression analysis of the association between anti-Hsp60 and anti-Hsp70 with risk for asthma

Variables*	Adjusted Regression coefficient	Standard error	*χ*^2 ^Value	*P *value	OR (95% CI)**
Constant	-0.977	0.509	3.676	0.055	
Sex	0.392	0.318	1.519	0.218	1.48 (0.79~2.76)
Age	0.017	0.109	0.026	0.872	1.02 (0.82~1.26)
Family history	0.602	0.219	8.215	0.085	2.01 (1.25~3.25)
Anti-Hsp60	0.459	0.328	1.965	0.161	1.58 (0.83~3.01)
Anti-Hsp70	0.794	0.248	10.224	0.001	2.21 (1.35~3.59)

*The dependent variable is the status of asthmas patient or control; the independent variables included Sex: 0 = male and 1 = female; Age: continuous variable in years; Family history: 0 = no and 1 = yes; Anti-Hsp60: 0 = negative and 1 = positive; Anti-Hsp70: 0 = negative and 1 = positive.

**OR, odds ratio, and CI, confidence interval.

### Correlation of anti-Hsp60 and anti-Hsp70 with the severity of asthma

To understand the possible significance of the anti-Hsp60 and anti-Hsp70 in asthma, we analyzed the correlation of anti-Hsp60 and anti-Hsp70 with the severity of asthma. Table 4 shows that there was a significant increase of positive rates and dilutions of anti-Hsp60 and anti-Hsp70 as the severity of asthma increased. This table also shows that there were significantly positive correlations of anti-Hsp60 and anti-Hsp70 with the numerical categories of symptom severity (p < 0.05)

**Table 4:** Correlation of anti-Hsp70 and anti-Hsp60 with symptom severities

Symptom Severity	No.	Anti-Hsp70 No. (%)				Anti-Hsp60 No. (%)			
		1:10	1:20	1:40	1:80	1:10	1:20	1:40	1:80
Step1: intermittent	30	2 (6.7)	0 (0.0)	0 (0.0)	0 (0.0)	0 (0.0)	0 (0.0)	0 (0.0)	0 (0.0)
Step2: mild persistent	36	10 (27.8)	5 (13.9)	2 (5.6)	1 (2.8)	3 (8.3)	1 (2.8)	1 (2.8)	1 (2.8)
Step3&4 moderate & severe persistent**	29	20 (69.0)	14 (48.3)	11 (37.9)	8 27.6)	14 (48.3)	5 (17.2)	4 (14.0)	4 (14.0)
*R *value*		0.809	0.958	0.968	0.959	0.954	0.864	0.947	0.947
*P *value*		0.000	0.000	0.000	0.001	0.000	0.000	0.016	0.016

* The analyses of correlation of symptom severities with different dilutions of anti-Hsp70 and anti-Hsp60

**: there are two severe persistent patients with asthma

### Differences in the levels of IgE and IL-4 between asthma patients with positive and negative anti-Hsps

We finally compared the levels of IgE and IL-4, two important known risk factors for asthma, in the 95 asthma patients who were either positive (37 patients) or negative (58 patients) for the presence of anti-Hsp60 and anti-Hsp70. The patients positive for anti-Hsps were more likely than the antibody-negative group of patients to report a family history of asthma (48.6% Vs 13.8%, p < 0.001) and had higher concentrations of total IgE (758.2 Vs 313.9, *P *= 0.001) and IL-4 (36.9 Vs 28.5, p = 0.019) (Table 5). Further analysis showed that the presence of either anti-Hsp60 or anti-Hsp70 or both was significantly correlated with the levels of IgE and IL-4 in asthma patients (Table 6) (p < 0.05). These preliminary data also indicated a positive correlation between the presence of these autoantibodies and the severity of the disease (r = 0.461, p < 0.001 for anti-hsp60 and r = 0.538, p < 0.001 for anti-Hsp70) (Table 6) as well as a statistically significant correlation between anti-Hsp70 and anti-Hsp60 (r = 0.485, p < 0.001) by using the rank correlation analysis.

**Table 5:** Differences in selected risk factors, IgE, and IL-4 between asthma patients with positive and negative anti-Hsps

	Anti-Hsps(+) (n = 37)	Anti-Hsps(-) (n = 58)	*P *value
Sex (M/F)	23/14	31/27	0.403
Age (years, mean ± SD)	25.6 ± 15.6	29.3 ± 15.7	0.249
Family history (yes/no)	18/19	8/50	<0.001
IgE (IU/ml, mean ± SD)	758.2 ± 685.3	313.9 ± 458.2	0.001
IL-4 (ng/L, mean ± SD)	36.9 ± 17.2	28.5 ± 16.3	0.019

**Table 6:** Correlation between anti-Hsps, IgE, and IL-4 in 95 asthma patients

	IgE		IL-4		Anti-Hsp70		Anti-Hsp60		Anti-Hsps	
	
	*r*	*P*	*r*	*P*	*r*	*P*	*r*	*P*	*r*	*P*
IL-4	0.701	<0.001								
Anti-Hsp70	0.369	<0.001	0.222	0.010						
Anti-Hsp60	0.262	0.010	0.259	0.011	0.485	<0.001				
Anti-Hsps	0.366	<0.001	0.241	0.019	0.534	<0.001	0.814	<0.001		
Asthma severity	0.330	0.001	0.236	0.022	0.461	<0.001	0.538	<0.001	0.417	<0.001

## Discussion

The patients included in the present study had frequent asthmatic attacks, with signs of wheezing and higher levels of IgE and IL4 and low levels of FEV_1 _%Pre that are characteristics of asthma. We found that these asthma patients also had a significantly higher incidence of autoantibodies against combined Hsp60 and Hsp70 than the matched non-asthmatic controls and that, in particular, the presence of anti-Hsp70 was associated with asthma. Furthermore, there was a significant positive correlation between anti-Hsp60 and anti-Hsp70 and symptom severity of asthma. Thus among asthma patients, those who had positive anti-Hsp60 and anti-Hsp70 were more likely to report a family history of asthma and had higher levels of IgE and IL-4 than those without such antibodies. These findings provide evidence to support the hypothesis that the presence of anti-Hsp60 and especially anti-Hsp70 in asthma patients is strongly associated with asthma and the presence of these antibodies may predict symptom severity of asthma and provide new strategies for diagnosis and perhaps treatment of this disease.

Asthma is an immune and inflammatory disease, arising from complex interactions among genetic and environmental factors including bacterial or viral infection [14,15]. The production of autoantibodies against Hsps may result from genetic factors, infection, denaturation and release of Hsps as a result of cell damage, and the presence of antigen-specific lymphocytes [22,23].

Hsps are often the target of humoral and T cell-mediated immune responses to infection and may provide a link between the immune response to infection and autoimmunity caused by T lymphocyte cross-reactivity among Hsps of different origins [8,27,28]. It remains to be determined whether there is a relationship between the induction of Hsp70 and production of plasma autoantibodies against this Hsp and whether there is a cross-response of induced Hsps and autoantibodies against Hsps before and during the development of asthma. However, there are several lines of evidence that support an association between anti-Hsp60 and anti-Hsp70 and symptom severity in asthma patients. Firstly, as molecular chaperones, Hsps facilitate the synthesis, folding, assembly and intracellular trafficking of many functional proteins [3,29] and protect cells and organs against different types of damages [30,31] as observed in transient protection from ischemic injury in whole organs such as heart, brain and kidney [31-34]. Hsp70 has also been suggested to play an autoprotective role in asthma and lung injury [35-37]. Secondly, autoantibodies against Hsps may have significant roles in the pathogenesis and prognosis of diseases. For example, Shinghai et al reported the presence of antibodies against Hsps in patients with autoimmune liver diseases and suggested that the presence of anti-Hsp70 was an indicator for the disease activity of primary biliary cirrhosis [8]. Earlier results from our lab also suggested that the presence of such antibodies might help assess if workers are experiencing abnormal stress within their living and working environment [21-23]. Xu et al and Schett et al have shown that mycobacterial Hsp65 may serve as an antigen to instigate chronic immune responses characteristic of human atherosclerosis. These antibodies were sustained among patients with the most severe degree of underlying atherosclerosis and were demonstrated to predict 5-year mortality [11,12,20]. Thirdly, enhanced expression of Hsp70 has been detected in bronchi and alveolar macrophages of patients with asthma and correlated with intrapulmonary eosinophilia, airway inflammation, hyperresponsiveness of bronchi [38], and severity of the disease [14,35,39]. A cross-response of induced plasma and cellular Hsps and autoantibodies against Hsps in human, has been suggested to play a role in the development and prognosis of atherosclerosis [40,41]. However, it is still unknown whether there is a cross-response between the induction of Hsps in bronchi of patients with asthma and the presence of anti-Hsps and its biological effects.

The development of most immune diseases depends on the cytokines interleukin-2 and interferon-γ produced by type 1 helper T cells (Th1), whereas the development of allergic diseases requires IL-4 and IL-5, both of which are produced by type 2 helper T cells (Th2). The reciprocal down-regulation of Th1 cells by Th2 cytokines raises the possibility that these cytokines are involved in allergy or immunity [42]. IL-4 is one of the first signals for a switch to the synthesis of IgE and IL-4 binds to receptors on B cells to induce and amplify the synthesis of IgE [43]. There is a cross-linking of IgE with allergens to activate a series of response seen in asthma [44]. Epidemiological and clinical observations have linked IgE antibodies to the severity of asthma and the initial and sustained responses of the airways to allergens [45,46]. At this time, the molecular events that link antibody to Hsp70 to the production of IL-4 and IgE and the interaction among these factors in patients with asthma remain to be investigated.

## Conclusions

The present study showed that there was a significant increase in positive rates of antibodies against Hsp60 and Hsp70 in patients with asthma and that the presence of autoantibodies against Hsp70 was associated with the severity of asthma. The presence of anti-Hsp70 associated with a high risk of asthma was also correlated with the family history of asthma and higher levels of total IgE and IL-4 in the patients. These results suggest that anti-Hsp70 correlate with the pathogenesis of asthma, but the precise underlying molecular mechanisms for these interactions remain to be established.

## Authors' contributions

MY and LC performed the immunoblot assays, the acquisition of data and wrote the first draft of the manuscript. FW carried out the collection and statistical analysis of data. QW was responsible for the analysis and interpretation of data, and critical revision of the manuscript. TW and RMT initiated the project, designed the experiments, and wrote the manuscript.
